# Early onset bilateral juvenile myasthenia gravis masquerading as simple congenital ptosis

**DOI:** 10.3205/oc000058

**Published:** 2017-03-07

**Authors:** Md. Shahid Alam, Pratheeba Devi Nivean

**Affiliations:** 1 Department of Orbit, Oculoplasty, Reconstructive and Aesthetic services, Sankara Nethralaya, Medical Research Foundation, Chennai, Tamil Nadu, India; 2 M.N. Eye Hospital, Chennai, India

**Keywords:** juvenile myasthenia, childhood myasthenia, ocular myasthenic syndrome, masquerade, congenital ptosis

## Abstract

Myasthenia gravis is an autoimmune disorder affecting the neuromuscular junction. Ocular myasthenia gravis presents as ptosis with extraocular motility restriction and is prone to be misdiagnosed as third nerve palsy or congenital or aponeurotic ptosis. Juvenile ocular myasthenia gravis in very young children is difficult to diagnose and can be easily labeled as a case of congenital ptosis, the more so when the condition is bilateral. We present a case of a two-year-old child who presented with bilateral ptosis and was diagnosed as a case of simple congenital ptosis elsewhere with the advice to undergo tarsofrontalis sling surgery. The child was diagnosed with juvenile myasthenia gravis on thorough history, examination, and systemic evaluation and was started on anti-myasthenic treatment.

## Introduction

Myasthenia gravis is the most common disorder of neuromuscular junction with an estimated prevalence of 20 cases per 100,000 population [1]. The disease is caused by antibodies directed to various proteins at the neuromuscular junction, most commonly the acetyl choline receptors. Ocular weakness is the most common complaint presenting in over half of the patients [1], [2]. This leads to variable weakness of various extraocular muscles including levator palpebrae superioris and orbicularis oculi [3]. Childhood ocular myasthenia is a rare entity which is prone to be misdiagnosed [4]. Patients can present with ptosis, strabismus, and diplopia. Early onset childhood ocular myasthenia is even rarer and poses a diagnostic challenge to the clinician.

We herewith report a rare case of bilateral juvenile myasthenia gravis in a 2-year-old child who was misdiagnosed with simple congenital ptosis and was also advised surgery for the same.

## Case description

**Figure 1:**
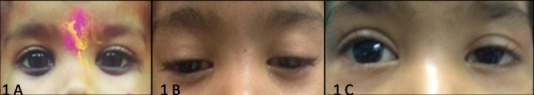
A: External photograph of the patient showing normal upper lid position in both eyes before onset of symptoms. B: External photograph of the patient showing bilateral severe ptosis. C: External photograph of the patient showing marked improvement after starting treatment for myasthenia gravis.

A 2-year-old male child presented to us with complaints of drooping of both upper lids for the past 5 months. The parents reported improvement of drooping after sleep. They also complained that the motility of the eye balls was limited and the child turned the head to fixate on objects. The child had fallen from height 5 months back and the parents were correlating the complaints to the trauma. The patient had already been examined at some other centre where he was diagnosed to have congenital ptosis and was advised to undergo tarsofrontalis sling surgery to clear the visual axis and prevent amblyopia. On examination, visual acuity by fixation method was central, steady and maintained (CSM) in both the eyes. There was not any evident amblyopia on the basis of fixation preference. The child was orthophoric on Hirschberg examination. Marginal reflex distance (MRD-1) was around 1 mm and was found to be variable. Extraocular movements were difficult to examine but were found to be limited to varying extents in all direction of gazes. Ice pack test was performed with much difficulty and mild improvement (around 1 mm) was noted. The rest of the anterior and posterior segment examination was within normal limits. Based on history and examination, a differential diagnosis of mitochondrial myopathy and juvenile myasthenia gravis was considered. Blood investigations revealed a highly raised serum acetyl choline receptor antibody level. The opinion of a neurologist was sought and the patient was started on pyridostigmine tablets. There was marked improvement in ptosis and extraocular movements following the initiation of therapy (Figure 1 (Fig. 1)). The patient was doing well on his follow-up after 2 years.

## Discussion

Myasthenia gravis is termed ocular myasthenia gravis (OMG) when weakness is limited exclusively to the eyelids and extraocular muscles [3]. There are 3 forms of childhood myasthenia. Neonatal, juvenile, and congenital myasthenic syndrome [4]. Neonatal myasthenia occurs because of transplacental transmission of autoantibodies from a mother to her child [5]. It is transient and the child recovers once the antibodies are cleared from the circulation. Congenital myasthenic syndrome is a heterogeneous group of genetically inherited disorders of neuromuscular junction [6].

Myasthenia gravis presenting before 19 years of age is termed juvenile myasthenia gravis [7]. Up to 50% of all cases of myasthenia in Chinese population present in childhood [8], while in Caucasians only 10% of the cases present with prepubertal onset [9].

In a study by VanderPluym et al. on clinical characteristics of pediatric myasthenia, almost 36% of the patients had ocular myasthenia and the age for the same ranged from 18 months to 11 years [10]. 8 out of 18 patients (44%) were under 3 years of age and 10 patients presented with bilateral ptosis [10]. The clinical features in the juvenile myasthenia group showed a predominance of generalized symptoms (65%) compared to exclusively ocular symptoms (35%).

In their report of 3 cases of juvenile myasthenia gravis, Gradient et al. found only a single case to be under 3 years of age and the case was not exclusively ocular [11].

In their article on juvenile myasthenia gravis with prepubertal onset, Evoli et al. found the age range to be between 1.5 and 9.2 years and only 5 patients had ocular myasthenia [12].

Myasthenia gravis is a great mimicker and resembles various neurological and muscular disorders. Early onset ocular juvenile myasthenia is prone to be misdiagnosed because the clinical features have quite a resemblance with congenital ptosis. The chance of misdiagnosis increases in bilateral cases as presented in the current case report.

The key to a correct diagnosis lies in thorough history and meticulous examination. Various bedside tests including ice test and Cogan lid twitch help in arriving at a correct diagnosis. Acetyl choline receptor (AchR) antibodies are present only in 55% of cases of ocular myasthenia [7]. Anti-MuSK (muscle-specific tyrosine kinase) antibodies are found positive in 70% of AchR-negative patients and these patients have a higher chance of developing generalized myasthenia or to go into myasthenic crisis [4].

Electrophysiological testing is invaluable in diagnosis of suspected juvenile myasthenia gravis. Repetitive nerve stimulation test shows a decremental response of >10% in the compound motor action potential by the 4^th^ or 5^th^ stimulation. 

Management of juvenile myasthenia gravis is based on similar principles as of adult myasthenia (anticholine esterase inhibitors, steroids and immunosuppressant) [7]. Since our patient showed dramatic response to pyridostigmine, addition of steroids was not considered. Prepubertal patients undergoing thymectomy have shown increased remission rates [13]. Prepubertal form of juvenile myasthenia gravis has better prognosis and less chances of developing generalized myasthenia compared to its postpubertal and adult counterpart [14]. Our case was well maintained on pyridostigmine at 2 years of follow-up without developing any symptoms of generalized myasthenia.

To conclude, the present case report highlights the importance of including myasthenia gravis in the differential diagnosis of any child presenting with ptosis with or without diplopia. A proper history and meticulous examination are the key to the diagnosis and better management in all such cases.

## Notes

### Competing interests

The authors declare that they have no competing interests.
